# Linkage analysis of alcohol dependence using both affected and discordant sib pairs

**DOI:** 10.1186/1471-2156-6-S1-S36

**Published:** 2005-12-30

**Authors:** Pei-Ying Shih, Tao Wang, Chao Xing, Moumita Sinha, Yeunjoo Song, Robert C Elston

**Affiliations:** 1 Department of Epidemiology & Biostatistics, Case Western Reserve University, 2103 Cornell Road, Wolstein Research Building, Cleveland, OH 44106 USA

## Abstract

The basic idea of affected-sib-pair (ASP) linkage analysis is to test whether the inheritance pattern of a marker deviates from Mendelian expectation in a sample of ASPs. The test depends on an assumed Mendelian control distribution of the number of marker alleles shared identical by descent (IBD), i.e., 1/4, 1/2, and 1/4 for 2, 1, and 0 allele(s) IBD, respectively. However, Mendelian transmission may not always hold, for example because of inbreeding or meiotic drive at the marker or a nearby locus. A more robust and valid approach is to incorporate discordant-sib-pairs (DSPs) as controls to avoid possible false-positive results. To be robust to deviation from Mendelian transmission, here we analyzed Collaborative Study on the Genetics of Alcoholism data by modifying the ASP LOD score method to contrast the estimated distribution of the number of allele(s) shared IBD by ASPs with that by DSPs, instead of with the expected distribution under the Mendelian assumption. This strategy assesses the difference in IBD sharing between ASPs and the IBD sharing between DSPs. Further, it works better than the conventional LOD score ASP linkage method in these data in the sense of avoiding false-positive linkage evidence.

## Background

Alcohol dependence (alcoholism) is a highly familial disorder that is a leading cause of morbidity and premature death. Several lines of evidence suggest a substantial genetic component to the risk for alcoholism [1]. The Collaborative Study of the Genetics of Alcoholism (COGA) is a 6-center program to detect and map susceptibility genes for alcoholism and related phenotypes. We report on the results of COGA data to identify susceptibility loci for alcohol dependence. Affected-sib-pair (ASP) linkage analysis was performed to detect susceptibility loci. ASP linkage analysis has been one of the most popular linkage methods used since Risch [2] introduced a LOD score formulation for it. In principle, the basic idea of this linkage method is to find those chromosomal regions that tend to be shared excessively between affected sibs [3]. ASP linkage analysis tests whether the inheritance pattern of a marker deviates from Mendelian expectation of independent segregation of alleles in a sample of ASPs. The test depends on a control distribution of the number of marker alleles shared identical by descent (IBD), i.e., 1/4, 1/2, and 1/4 for sharing 2, 1, and 0 allele(s) IBD, respectively. That is, the test focuses on searching for the chromosomal locations with excessive allele sharing between ASPs compared to Mendelian transmission. However, searching for chromosomal regions with excessive allele sharing among ASPs does not exclusively indicate evidence for linkage, because several phenomena other than linkage, such as inbreeding (when parental information is not available) or meiotic drive at the marker or nearby loci (when survival selection exists on the same chromosome), will also cause excess allele sharing. Hence, any linkage method that uses ASPs alone may produce a false-positive linkage signal, because of using a biased null IBD distribution under the Mendelian assumption as a control. A robust approach is to incorporate discordant-sib-pairs (DSP) as a control to avoid possible false-positive results. This is based on the fact that, in a region where potential deviation from Mendelian inheritance arises in the absence of linkage, the number of alleles shared by DSPs should be no less than the number of alleles shared by ASPs. Specifically, a test statistic that incorporates DSP information in addition to ASP information would be less sensitive to deviation from the Mendelian distribution when there is no linkage and, hence, would avoid false-positive linkage signals on that account. To attain this goal, here we analyzed COGA data by modifying the LOD score ASP method as implemented in the S.A.G.E. [4] program LODPAL, which uses the conditional logistic model [5], to use the estimated distribution of the number of allele(s) shared IBD by DSPs as a control instead of the expected distribution under the Mendelian assumption.

## Methods

### Data used

In this analysis, 315 microsatellite markers located on the autosomal chromosomes were used in the genome scan. Affected sibs were defined to be sibs who met both DSM-III-R Alcohol Dependence and Feighner definite alcoholism criteria (i.e., those who were coded as 'affected' in the ALDX1 variable), and unaffected sibs were defined to be "pure" unaffected. Sibs who reported some symptoms but did not meet the diagnostic criteria, or who had never consumed alcohol, were assumed to have unknown phenotypes.

### Linkage methods

First we conducted a 2-cM genome scan using the ASP method with and without constraints [6] as implemented in LODPAL (both the '2-parameter' and '1-parameter' options were performed for each constraint condition). For the same number of parameters in a model (1 or 2), the model with constraints and the model without constraints gave a similar pattern of linkage evidence. To be conservative, the linkage signals obtained from the 1-parameter model with constraints were subjected to further analysis. We examined the IBD distribution for ASPs and DSPs at suspected regions targeted by the preceding ASP analysis. If there was a linkage between a disease locus and markers in a specific region, we should expect there to be some discrepancy between the IBD sharing distributions for both ASPs and DSPs (in an opposite direction). If the IBD distributions of ASPs and DSPs are similar, even though they may deviate from the IBD distribution expected under Mendelian inheritance, the region targeted by the ASP method could well not really be linked to a disease locus and the linkage signal might just be due to an invalid "control". To correct this bias, we modified the likelihood ratio (LR) used for an ASP analysis in LODPAL by simply using an estimated IBD distribution from DSPs instead of the "expected IBD distribution" under the null hypothesis of no linkage. The likelihood ratio is thus given by the product, over all sib pairs, of



where λ_i _is the relative risk to an individual who shares *i *allele(s) IBD with an affected sib; and  and  are the IBD distributions estimated at a given location from the observed marker data for ASPs and DSPs, respectively. With the DSPs' IBD sharing distribution serving as a control based on the above rationale, the modified LR statistic will be close to one under the null hypothesis of no linkage whether or not there is overall deviation from Mendelian inheritance, so that the false-positive linkage signals would be reduced. Finally, we compared the results of this new ASP/DSP method with those from the original Haseman-Elston (HE) regression analysis [7], giving affected individuals a quantitative score of 1 and unaffected individuals a quantitative score of 0 [8], as implemented in the S.A.G.E. [4] program SIBPAL, which allows for dependencies between sib pairs in the same family [9]. Because this HE regression also includes both ASP and DSP information and tests the correlation between phenotypic similarity and genotypic similarity described by the marker IBD, it should be robust to deviation from Mendelian assumptions. Here, in order to make results from LODPAL and SIBPAL comparable, we rescaled each LOD score in LODPAL into pP = -log_10_(*p*-value), with the *p*-value corresponding to the LOD score, i.e., computed by assuming the asymptotic chi-squared distribution with 1 d.f. for the 1-parameter model.

## Results

**Figure 1:**
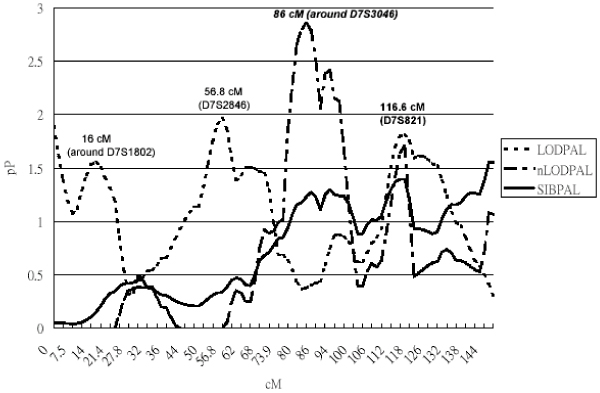
Plots of pP from LODPAL, nLODPAL, and SIBPAL, chromosome 7.

**Figure 2:**
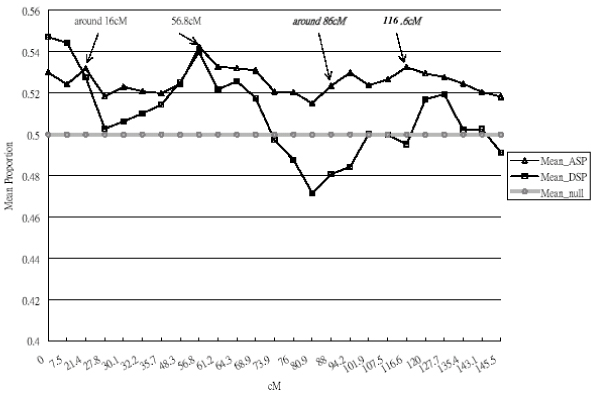
Mean proportion of alleles shared (0*f_0_+(0.5)*f1+1*f2) by ASPs and DSPs, chromosome 7.

We first conducted a genome-wide linkage scan by the ASP method (results not shown here) and any linkage signal close to 0 cM or the q end of the chromosome was ignored, because in multipoint linkage analysis IBD estimation around these 2 points is often unstable, with the result that the corresponding linkage information would not be reliable. This indicated 3 peaks on chromosome 7 (see dotted line in Figure 1). The 3 peaks were at 16 cM (around the marker D7S1802), 56.8 cM (marker D7S2846), and 116.6 cM (marker D7S821). The IBD distributions of the ASPs and DSPs were examined across all markers on chromosome 7 (data not shown here). We found the IBD distributions to be similar for both ASPs and DSPs at the peaks around 16 cM (D7S1802) and 56.8 cM (D7S2846), suggesting the 2 peaks are false-positive signals (Table 1). We further analyzed the data of chromosome 7 by the new LODPAL (nLODPAL), which used the modification indicated above, and by HE regression (using the option "diff" in the S.A.G.E. program SIBPAL [9,10]) (Figure 1). The results showed that the 2 suspected false signals were not detected by our modified LODPAL or HE regression. The signal around 116.6 cM (D7S821) was detected by the original ASP method, HE regression, and our modification. A new signal was detected in the region at about 86 cM (around marker D7S3046) by both our modification and HE regression. The findings from both our modification and HE regression that peaks at 16 cM and 56.8 cM disappear, while a new peak at 86 cM appears, are consistent with the information seen in the IBD distributions of the ASPs and DSPs. Examining the IBD distribution of the ASPs alone, the first 2 locations show relatively larger deviations from the null IBD distribution of 0.25, compared to the third location, so that linkage signals at the first 2 locations were identified by ASPs-based LODPAL (see the second and third columns in Table 1). On the other hand, incorporating the IBD distribution of the DSPs, only the third location showed quite a large difference in IBD distributions between ASPs and DSPs (see the sixth and seventh columns in Table 1), and thus only this new peak at 86 cM appears in ASP/DSP-based nLODPAL and SIBPAL. In addition, Figure 2 shows the pattern of the mean proportion of allele sharing among ASPs and DSPs across all markers on chromosome 7. Overall, the pattern shown in Figure 2 is compatible with the linkage evidence shown in our modified LODPAL, where a larger difference in mean proportions of allele sharing between ASPs and DSPs corresponds to a stronger linkage signal. Examining the mean proportion for ASPs alone, the 3 locations with the 3 largest departures from null mean proportion of 0.5 are D7S2846 (56.8 cM), D7S821 (116.6 cM), and D7S1802 (16 cM), which are reasonably identified by the original ASP method. However, when mean proportions for ASPs and DSPs were assessed simultaneously, D7S2846 (56.8 cM) and D7S1802 (16 cM) gave only a tiny deviation of IBD sharing between ASPs and DSPs, which suggests false linkage evidence at these 2 regions.

## Discussion

Previous study [1] of the COGA data using an ASP linkage method showed highly suggestive evidence of linkage on chromosomes 1 and 7, and more modest evidence on chromosome 2. In this analysis, we conducted a similar genome scan but using a modified ASP/DSP method. Because the original ASP method is based on the assumption of Mendelian transmission, the ASP statistic can be invalid when this assumption does not hold. To maintain the validity of the statistic, we propose to obtain a control from the data at hand, i.e., the data on DSPs. Using this modified statistic, the dissimilarity in trend of allele sharing between ASPs and DSPs is considered in order to prevent high LOD scores that can be false linkage signals. This modified ASP/DSP method worked better than the original ASP method with respect to avoiding probable false signals on chromosome 7. Not only was the signal at 116.6 cM detected, but also a new signal at 86 cM was detected that was also seen with HE regression. Both the modified ASP/DSP and HE regression make use of the ASP and DSP information in a similar manner, and may be more powerful than the original ASP method for this dataset. The main difference between these 2 methods of analysis lies in the fact that HE uses linear regression, whereas our new method uses non-linear regression: this has implications for the interpretation of any covariates that are included in the analysis [9]. The signals at 86 cM and 116.6 cM warrant further study.

## Abbreviations

ASP: Affected sib pair

COGA: Collaborative Study on the Genetics of Alcoholism

DSP: Discordant sib pairHE: Haseman-Elston

IBD: Identity by descent

LR: Likelihood ratio

## Authors' contributions

P-YS carried out the linkage analysis, participated in the genome scan using the LODPAL program and the identification of the linkage regions on chromosome 7 using the SIBPAL program, and drafted the manuscript. TW contributed in modification of the test statistic as implemented in LODPAL to the new test statistic as implemented in nLODPAL and drafted the initial manuscript presented at the GAW meeting. CX carried out the linkage analysis on chromosome 7 using nLODPAL. MS helped to obtain the estimated IBD distributions of sib pairs using the S.A.G.E. program GENEIBD. YS developed the program nLODPAL. RCE conceived the study, participated in its design and coordination, and helped to draft the manuscript.

## Figures and Tables

**Table 1:** The distribution of IBD sharing for ASPs and DSPs and their difference (in absolute value) at three targeted markers

Marker	ASPs		DSPs		| ASPs-DSPs |	
			
						
16 cM (around D7S1802)	0.279	0.230	0.304	0.215	0.025	0.015
56.8 cM (D7S2846)	0.284	0.199	0.295	0.215	0.011	0.016
86 cM (around D7S3046)	0.213	0.261	0.252	0.213	0.039	0.048
116.6 cM (D7S821)	0.300	0.235	0.259	0.268	0.041	0.033
